# SNP-Seek database of SNPs derived from 3000 rice genomes

**DOI:** 10.1093/nar/gku1039

**Published:** 2014-11-27

**Authors:** Nickolai Alexandrov, Shuaishuai Tai, Wensheng Wang, Locedie Mansueto, Kevin Palis, Roven Rommel Fuentes, Victor Jun Ulat, Dmytro Chebotarov, Gengyun Zhang, Zhikang Li, Ramil Mauleon, Ruaraidh Sackville Hamilton, Kenneth L. McNally

**Affiliations:** 1 T.T.Chang Genetic Resources Center, IRRI, Los Baños, Laguna 4031, Philippines; 2 BGI, Shenzhen 518083, China; 3 Institute of Crop Sciences/National Key Facility for Crop Gene Resources and Genetic Improvement, Chinese Academy of Agricultural Sciences, Beijing, 100081

## Abstract

We have identified about 20 million rice SNPs by aligning reads from the 3000 rice genomes project with the Nipponbare genome. The SNPs and allele information are organized into a SNP-Seek system (http://www.oryzasnp.org/iric-portal/), which consists of Oracle database having a total number of rows with SNP genotypes close to 60 billion (20 M SNPs × 3 K rice lines) and web interface for convenient querying. The database allows quick retrieving of SNP alleles for all varieties in a given genome region, finding different alleles from predefined varieties and querying basic passport and morphological phenotypic information about sequenced rice lines. SNPs can be visualized together with the gene structures in JBrowse genome browser. Evolutionary relationships between rice varieties can be explored using phylogenetic trees or multidimensional scaling plots.

## INTRODUCTION

The current rate of increasing rice yield by traditional breeding is insufficient to feed the growing population in the near future (1). The observed trends in climate change and air pollution create even bigger threats to the global food supply (2). A promising solution to this problem can be the application of modern molecular breeding technologies to ongoing rice breeding programs. This approach has been utilized to increase disease resistance, drought tolerance and other agronomically important traits (3–5). Understanding the differences in genome structures, combined with phenotyping observations, gene expression and other information, is an important step toward establishing gene-trait associations, building predictive models and applying these models in the breeding process. The 3000 rice genome project (6) produced millions of genomic reads for a diverse set of rice varieties. SNP-Seek database is designed to provide a user-friendly access to the single nucleotide polymorphisms, or SNPs, identified from this data. Short, 83 bp pair-ended Illumina reads were aligned using the BWA program (7) to the Nipponbare temperate japonica genome assembly (8), resulting in average of 14× coverage of rice genome among all the varieties. SNP calls were made using GATK pipeline (9) as described in (6).

## SNP DATA

**Figure 1.**
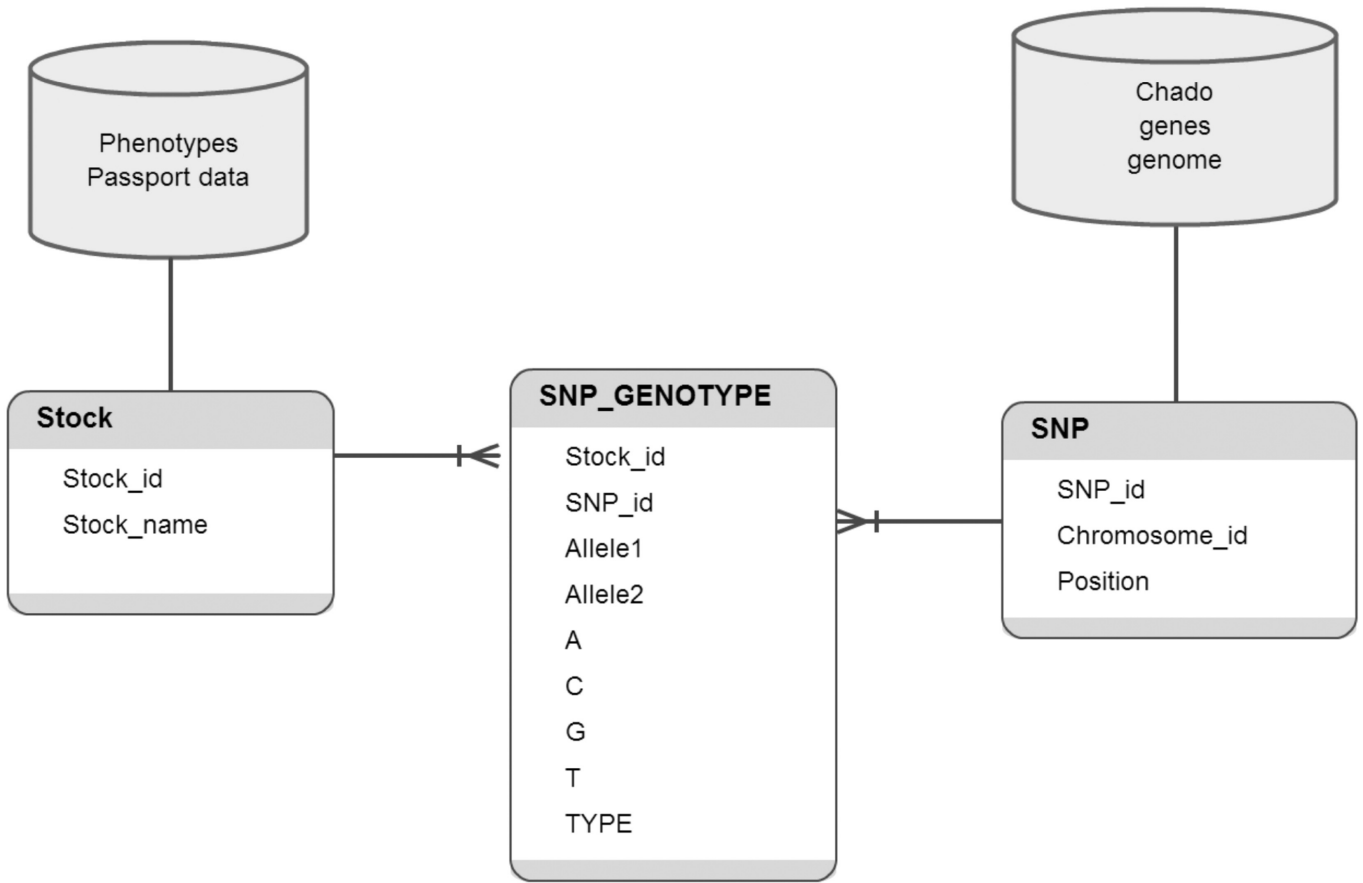
Basic schema of the SNP-Seek database

**Figure 2.**
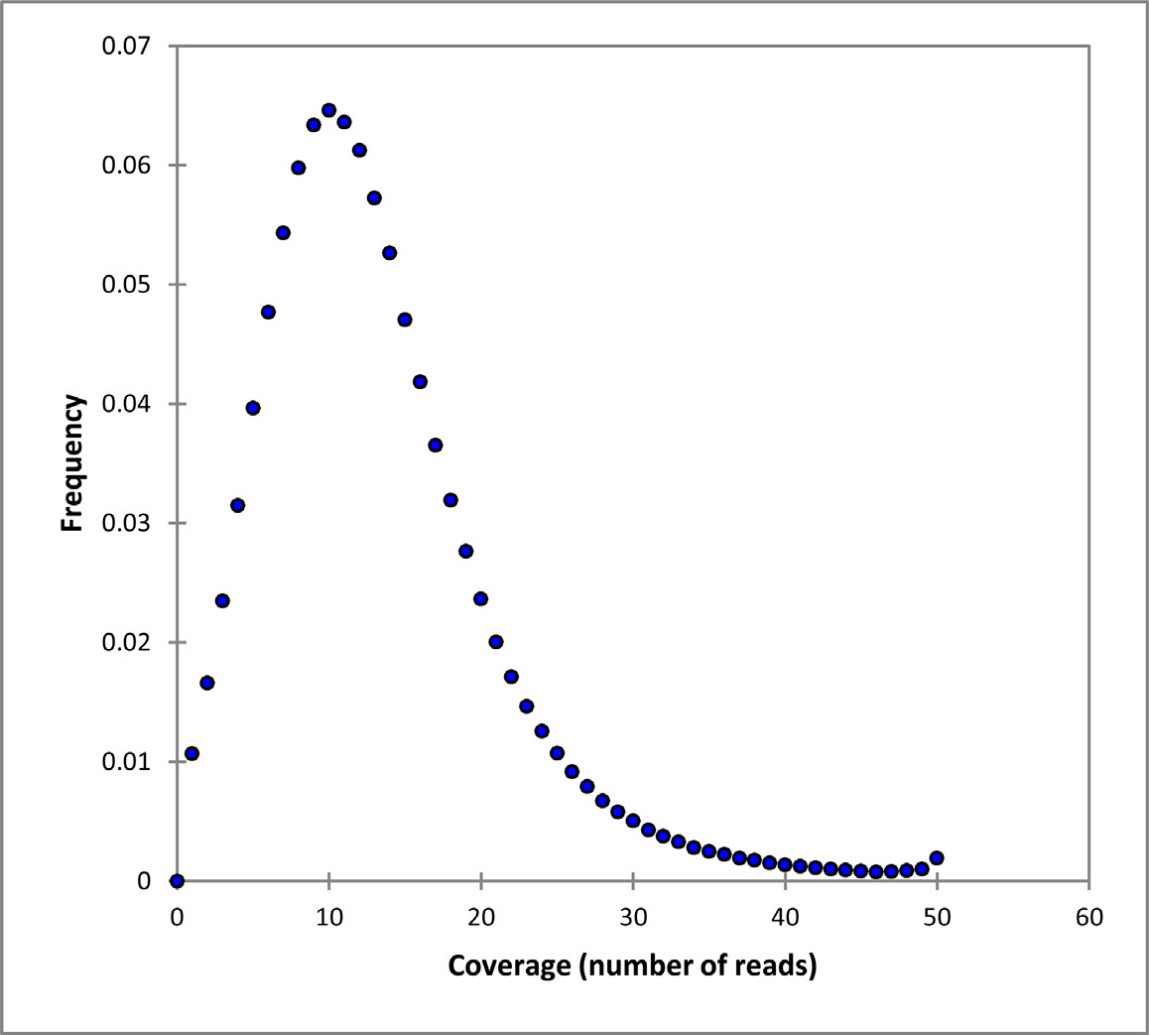
Distribution of SNP coverage

For the SNP-Seek database we have considered only SNPs, ignoring indels. A union of all SNPs extracted from 3000 vcf files consists of 23 M SNPs. To eliminate potentially false SNPs, we have collected only SNPs that have the minor allele in at least two different varieties. The number of such SNPs is 20 M. All the genotype calls at these positions were combined into one file of ∼20 M × 3 K SNP calls, and the data were loaded into an Oracle schema using three main tables: STOCK, SNP and SNP_GENOTYPE (Figure 1). Some varieties lack reads mapping to the SNP position, and for them no SNP calls were recorded. Distribution of the SNP coverage is shown in Figure 2. About 90% of all SNP calls have a number of supporting reads greater than or equal to four. Out of them, 98% have a major allele frequency >90% and are considered to be homozygous, 1.1% have two alleles with frequencies between 40 and 60% and considered to be heterozygous, and the remaining 0.9% represent other cases when the SNP could not be classified as neither heterozygous nor homozygous. More than 98% of SNPs have exactly two different allelic variants in 3000 varieties, 1.7% of SNPs have three variants and 0.02% of SNPs have all four nucleotides in different genomes mapped to that SNP position. There are 2.3× more transitions than transvertions in our database (Table 1).

**Table 1.** Types of allele variants and their frequencies in rice SNPs

Allele variants	Frequency,%
A/G + C/T	70
A/C + G/T	15
A/T	9
C/G	6

**Figure 3.**
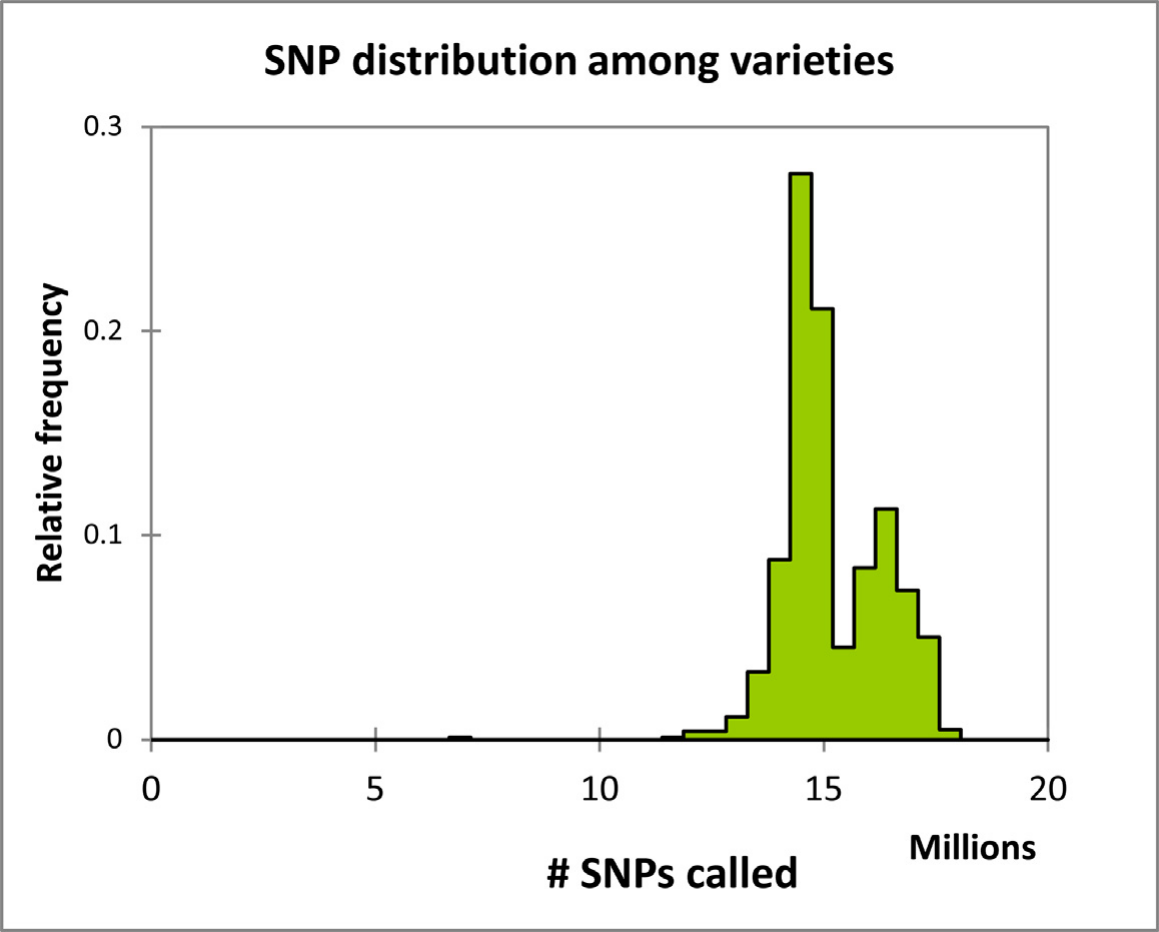
SNP distribution by varieties. The major peak shows that about 14 M SNPs have been called in most varieties. The bimodal plot indicates that a fraction of SNPs are missing in some varieties, likely due to lack of mapped reads in variable regions.

Not all SNPs have been called in all varieties. Actually, the distribution of the called SNPs among varieties is bimodal, with one mode at about 18 M SNP calls corresponding to japonica varieties which are close to the reference genome, and the second peak at about 14 M corresponding to the other varieties (Figure 3).

## GENOME ANNOTATION DATA

We used CHADO database schema (10) to store the Nipponbare reference genome and gene annotation, downloaded from the MSU rice web site (http://rice.plantbiology.msu.edu/) (8). To browse and visualize genes and SNPs in the rice genome, we integrated the JBrowse genome browser (11) as a feature of our site.

## PASSPORT AND MORPHOLOGICAL DATA

Most of the 3000 varieties (and eventually all) are conserved in the International Rice genebank housed at IRRI (12). Passport and basic morphological data from the source accession for the purified genetic stock are accessible via SNP-Seek.

## INTERFACES

**Figure 4.**
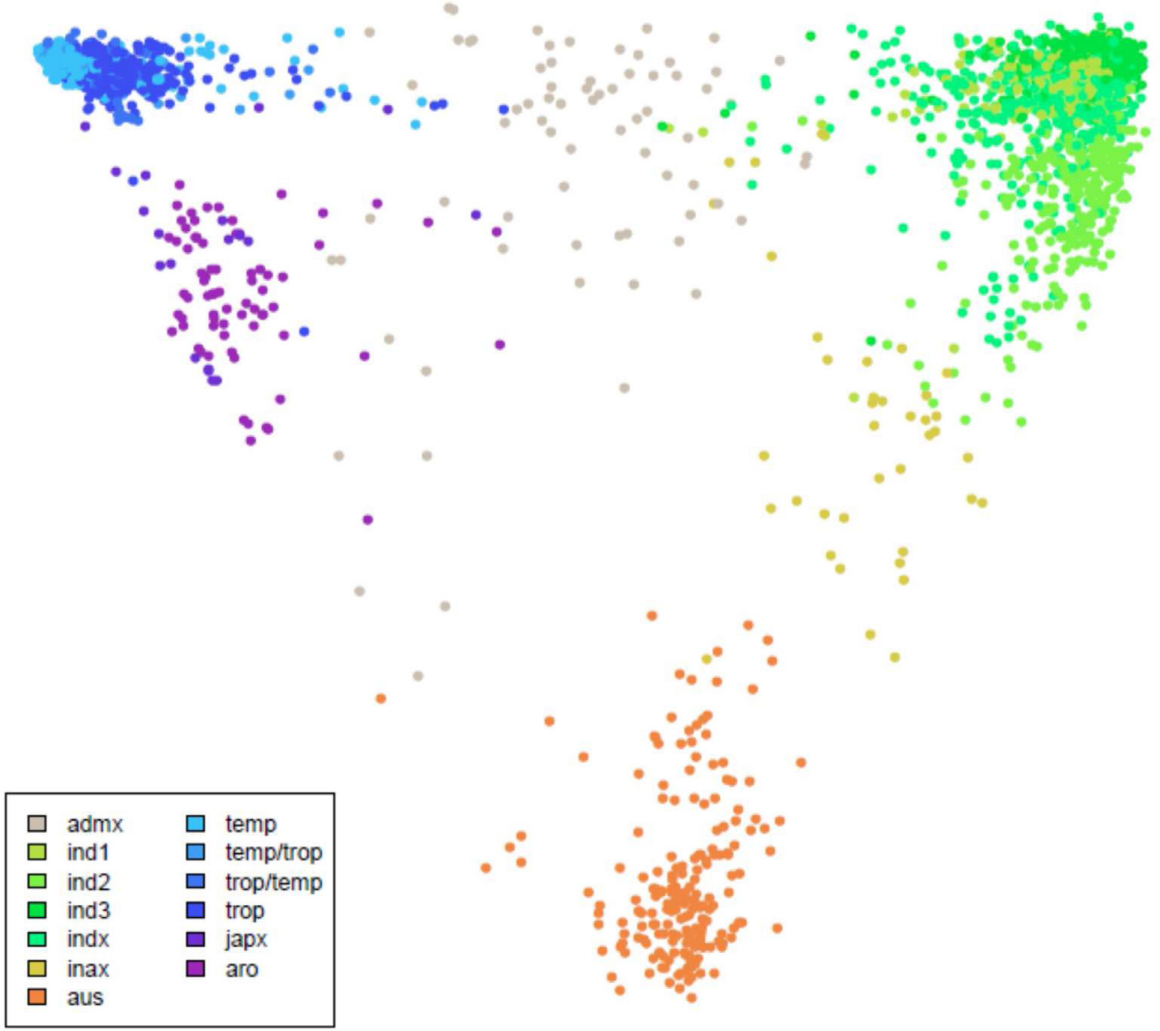
Multidimensional scaling plot of the 3000 rice varieties. Ind1, ind2 and ind3 are three groups of indica rice, indx corresponds to other indica varieties, temp is temperate japonica, trop is tropical japonica, temp/trop and trop/temp are admixed temperate and tropical japonica varieties, japx is other japonica varieties, aus is aus, inax is admixed aus and indica, aro is aromatic and admix is all other unassigned varieties.

We deployed interfaces to facilitate the following major types of queries: (i) for two varieties find all SNPs from a gene or genomic region that differentiate them; (ii) for a gene or genome region, show all SNP calls for all varieties (Supplementary Figure S1); (iii) find all sequenced varieties from a certain country or a subpopulation, which can be viewed as a phylogenetic tree, built using TreeConstructor class from BioJava (13) and rendered using jsPhyloSVG JavaScript library (14) (Supplementary Figure S2) or as a multidimensional scaling plots (Figure 4). The results of SNP search can be viewed as a table exported to text files, or visualized in JBrowse.

## USE CASE EXAMPLE FOR QUERYING A REGION OF INTEREST

**Figure 5.**
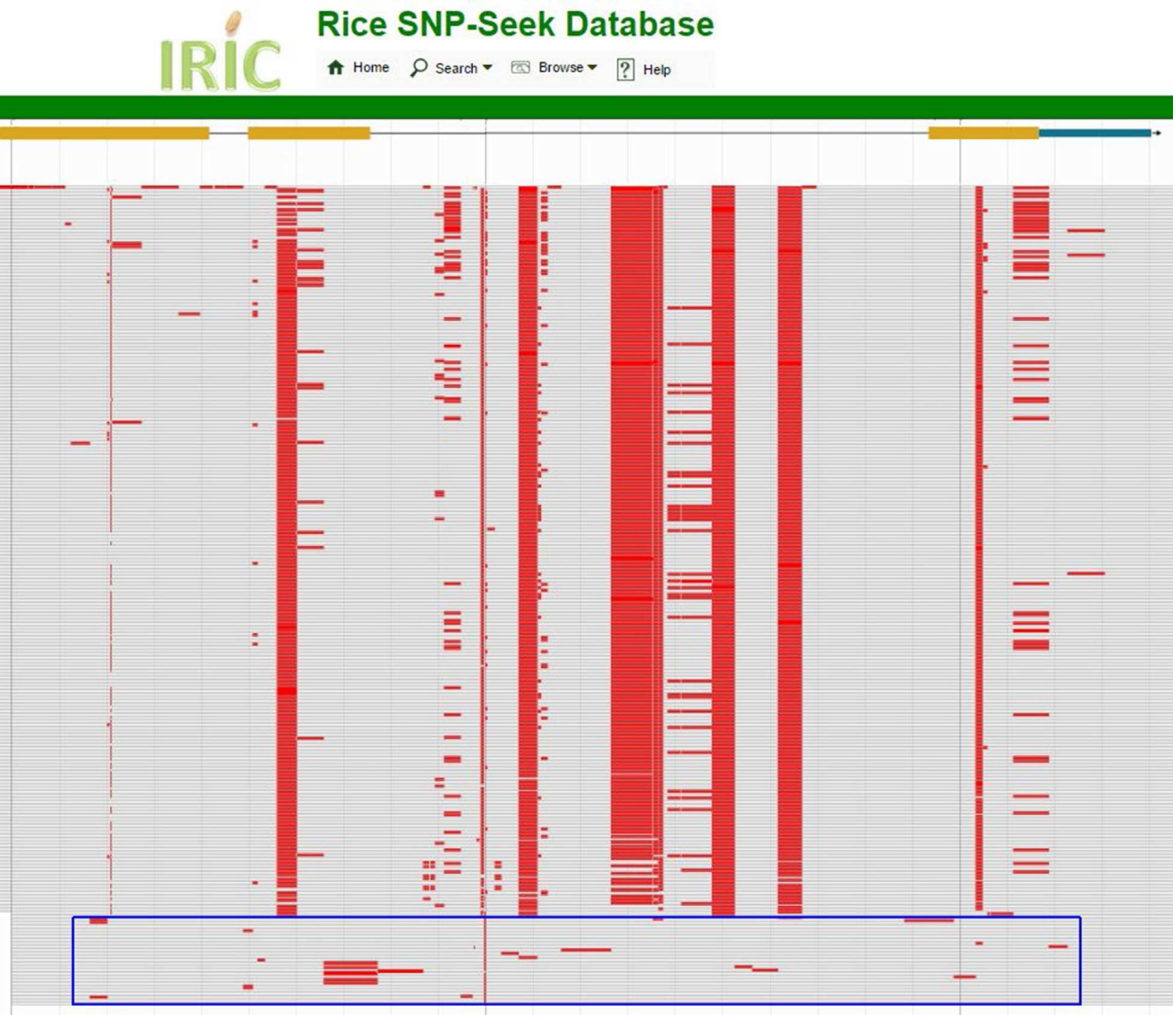
Jbrowse view of the SNP genotypes within the sd-1 gene (each variety is one row). Red blocks indicate polymorphism of the variety against Nipponbare. Shared SNP blocks are seen as vertical columns in red. The blue rectangle box in the bottom contains varieties that do not have these blocks.

We used Rice SNP-Seek database to quickly examine the diversity of the entire panel at a particular region of interest. We chose the sd-1 gene as test case due to its scientific importance in rice breeding. This semi-dwarf locus, causing a semi-dwarf stature of rice, was discovered by three different research groups to be a spontaneous mutation of GA 20-oxidase (formally named sd-1 gene), originating from the Taiwanese indica variety Deo-woo-gen. Its incorporation into IR8 and other varieties by rice breeding programs spurred the First Green Revolution in rice production in the late 1960s (15). Sd-1 is annotated in the Nipponbare genome by Michigan State University's Rice Genome Annotation Project as LOC_Os01g66100, on chromosome 1 from position 38 382 382 to 38 385 504 base pairs. On the home page of SNP-Seek, the <Genotype> module was opened and the coordinates of sd-1 were used to define the region to retrieve all SNPs, with <All Varieties> checked to select from all the varieties. Clicking on <Search> button resulted in the identification of 80 SNP positions (Supplementary Figure S1). An overall view of the SNP positions in the polymorphic panel shows at least eight distinct SNP blocks (Figure 5). In this particular panel group of mostly temperate japonica, two distinct SNP blocks can be seen as shared (Figure 5). Variety information can be obtained by typing the name of the varieties you see on the genome browser into the <Variety name> field of the Variety module. This use case is one of the examples detailed in the <Help> module.

## CONCLUSION

We have organized the largest collection of rice SNPs into the database data structures for convenient querying and provided user-friendly interfaces to find SNPs in certain genome regions. We have demonstrated that about 60 billion data points can be loaded into an Oracle database and queried with a reasonable (quick) response times. Most of the varieties in SNP-Seek database have passport and basic phenotypic data inherited from their source accession enabling genome-wide or gene-specific tests of association. The database is quickly developing and will be expanding in the near future to include short indels, larger structural variations, SNPs calls using other rice reference genomes.

## SUPPLEMENTARY DATA

Supplementary Data are available at NAR Online.
